# Editorial: Spatial epidemiology

**DOI:** 10.3389/fpubh.2024.1522631

**Published:** 2025-01-24

**Authors:** Chen Li, Kai Zhang, Shujuan Yang, Peng Jia

**Affiliations:** ^1^School of Resource and Environmental Sciences, Wuhan University, Wuhan, China; ^2^International Institute of Spatial Lifecourse Health (ISLE), Wuhan University, Wuhan, China; ^3^Department of Environmental Health Sciences, College of Integrated Health Sciences, University at Albany, State University of New York, Rensselaer, NY, United States; ^4^West China School of Public Health and West China Fourth Hospital, Sichuan University, Chengdu, China; ^5^School of Public Health, Wuhan University, Wuhan, China; ^6^Renmin Hospital (First School of Clinical Medicine), Wuhan University, Wuhan, China

**Keywords:** spatial epidemiology, chronic disease, infectious disease, environmental epidemiology, environmental exposure

Spatial epidemiology is a branch of epidemiology that focuses on the analysis of geographic variations in disease with respect to the distribution of demographic, environmental, behavioral, and socioeconomic risk factors (1). It involves analyzing spatial data to identify spatial clusters of health events, understand the influence of place and space on health outcomes, and assess the effectiveness of public health interventions (2). Recent advances in data availability and analytical methods have created new opportunities to improve research on both chronic non-communicable and infectious diseases.

## Chronic non-communicable diseases

With the rapid development of spatial technology, research exploring determinants of chronic non-communicable diseases has been not only on traditional non-spatial factors (e.g., lifestyle behaviors), but increasingly on spatial factors (e.g., natural and built environments) (3–5). We can now collect multi-source data about various types of environments with unprecedented precision and breadth, for example, from air quality monitoring stations, remote sensing images, social media carrying public opinions, and wearable sensors, which have formed a rich, complex information network. For example, a recent U.S. study estimated the negative health impacts of PM_2.5_ in Texas by using a software that, developed by the U.S. Environmental Protection Agency (EPA), uses a variety of exposure-response functions to integrate air pollutant, population, disease, and death data for health effect estimation at fine spatial scales (Bryan and Landrigan). This study found that, although the levels of PM_2.5_ concentration across most of the states complied with the EPA standards, at least 4.3% of the statewide premature deaths in 2016 could be attributed to PM_2.5_; moreover, PM_2.5_ was positively associated with the mortality of stroke, low birth weight, non-fatal lung cancers, Alzheimer's disease, and asthma. A major limitation is the uncertainty in the estimation of PM_2.5_ concentrations for regions with insufficient PM_2.5_ monitoring stations, which implies the importance and necessity of improving the existing modeling approaches for estimating the concentration of and exposure to PM_2.5_ with as high accuracy as possible (6).

Public health event monitoring tools on the basis of Internet search data are another set of popular, also low-cost, spatial epidemiological methods worthy of investigation. For example, the Baidu Index can quantify the search volume of specific keywords in a certain geographical area within a given time range, with a higher value indicating a higher level of popularity for the keyword. A recent Chinese study used the Baidu Index and data from the China's Health Statistical Yearbook to generate the heatmaps and estimate the national prevalence of asthma from 2011 to 2020, with the assumption that a higher Baidu Index value for the keyword “asthma” reflects a greater public concern and interest in asthma (Li et al.). Based on the Baidu Index and discharged records of 1,733,515 asthma patients, this study investigated the prevalence of asthma and its variations in the incidence across different regions. The prevalence of asthma from 2011 to 2020 and the hospitalization expenses for asthma increased while the length of hospital stay decreased. Although the asthma data were not actual real-world data, this study still provided an objective method based on internet search data to reflect the regional asthma prevalence.

## Infectious diseases

Spatial heterogeneities of natural and socioeconomic factors may underlie the variations in the incidence of infectious diseases (7). Furthermore, some spatial factors, such as population density and human mobility, are also related to the transmission and early detection of infectious diseases, such as COVID-19 (8, 9). Hence, spatial epidemiology needs to consider new indicators and methods for reducing inaccurate estimation and prediction. For example, a recent U.S. study collected daily PM_2.5_ concentrations and daily numbers of COVID-19 cases in 49 states during 2020–2021, to analyze temporal changes in the association between PM_2.5_ and COVID-19 incidence and the variations of the association across states, by combining a time-varying time-series generalized additive model with a Leroux-conditional-autoregression (LCAR) (Liu et al.). This study found that each 1 μg/m^3^ increase in the daily PM_2.5_ concentration was associated with a 0.92% increase in COVID-19 incidence, which exhibited significant spatiotemporal heterogeneities with stronger associations in the eastern and middle regions and with a *U*-shaped temporal change. Another Spanish study used six human mobility datasets (two individual movement datasets from social media and four individual trip datasets from the Spanish Ministerio de Transportes) and three meteorological datasets from satellite data (temperature, humidity, and ultraviolet radiance), to analyze the joint effect of human mobility and meteorological factors on the spreading of COVID-19 in 48 provinces during August 2020 to March 2021 (Conesa et al.). This study found a significant joint effect of mobility and meteorological factors on the increasing incidence of COVID-19 cases, while neither mobility nor meteorological factors were significantly associated with COVID-19 incidence.

Similarly, a Chinese study of the 3,710,962 bacillary dysentery (BD) cases from China CDC estimated the short-term association between monthly average temperature (MAT) and BD, introducing an innovative approach to address spatial heterogeneities by leveraging spatial autocorrelation (Wang et al.). This study first used a generalized additive model to independently estimate the province-specific association between MAT and BD. Then, a Leroux-prior-based conditional autoregression strategy was applied to spatially smooth the association and characterize its spatial distribution, accurately estimating the short-term association between MAT and BD. Compared with the existing methods, this method could enhance the accuracy of estimation in multi-city studies by considering spatial autocorrelation. Moreover, they found that the relative risks of BD in western provinces of China was higher than those in eastern provinces, which could provide scientific evidence for allocating public health resources in the future. However, a major limitation of this study is that the association between MAT and BD could not be estimated on a fine temporal or spatial scale, which implies the importance and necessity of improving the current spatial methods for estimating the individual exposure assessment.

## Outlook for future studies

Spatial epidemiology offers a powerful lens for understanding the distribution and burdens of chronic non-communicable and infectious diseases, and recent studies have further advanced this field. As we move forward, continuous innovation and interdisciplinary collaboration remain essential in refining our understanding of environmental health dynamics and improving public health outcomes. Future spatial epidemiological studies should focus on integrating multi-source data and exploring new methods (e.g., artificial intelligence) to address the current challenges (10).

## References

[1] Jia P. Spatial lifecourse epidemiology. Lancet Planet Health. (2019) 3:e57–9. 10.1016/S2542-5196(18)30245-6 30797406

[2] Elliott P Wartenberg D. Spatial epidemiology: current approaches and future challenges. Environ Health Perspect. (2004) 112:998–1006. 10.1289/ehp.6735 15198920 PMC1247193

[3] Jia P Stein A. Using remote sensing technology to measure environmental determinants of non-communicable diseases. Int J Epidemiol. (2017) 46:1343–4. 10.1093/ije/dyw365 28338734

[4] Jia P Stein A James P Brownson RC Wu T Xiao Q. Earth observation: investigating noncommunicable diseases from space. Annu Rev Public Health. (2019) 40:85–104. 10.1146/annurev-publhealth-040218-043807 30633713

[5] Jia P Xue H Yin L Stein A Wang M Wang Y. Spatial technologies in obesity research: current applications and future promise. Trends Endocrinol Metab. (2019) 30:211–23. 10.1016/j.tem.2018.12.003 30712979

[6] Qin K Wang Z Dai S Li Y Li M Li C. et al. Spatiotemporal patterns of air pollutants over the epidemic course: a national study in China. Remote Sens. (2024) 16:1298. 10.3390/rs16071298

[7] Jia P Dong W Yang S Zhan Z Tu L Lai S. Spatial lifecourse epidemiology and infectious disease research. Trends Parasitol. (2020) 36:235–8. 10.1016/j.pt.2019.12.012 32044243 PMC7172117

[8] Yang S Pan X Zeng P Jia P. Spatial technologies to strengthen traditional testing for SARS-CoV-2. Trends Microbiol. (2021) 29:1055–7. 10.1016/j.tim.2021.03.003 33775546 PMC7955912

[9] Huang Y Yang S Zou Y Su J Wu C Zhong B. Spatiotemporal epidemiology of COVID-19 from an epidemic course perspective. Geospat Health. (2022) 17:1023. 10.4081/gh.2022.1023 35147015

[10] Jia P Lakerveld J Wu J Stein A Root ED Sabel CE. Top 10 research priorities in spatial lifecourse epidemiology. Environ Health Perspect. (2019) 127:074501. 10.1289/EHP4868 31271296 PMC6791465

